# Social support and (complex) posttraumatic stress symptom severity: does gender matter?

**DOI:** 10.1080/20008066.2024.2398921

**Published:** 2024-10-15

**Authors:** Natalia E. Fares-Otero, Tamsin H. Sharp, Shreelakshmi Karthikeyan, Stefanie R. Balle, Sarah M. Quaatz, Eduard Vieta, Fredrik Åhs, Antje-Kathrin Allgaier, Adrián Arévalo, Rahel Bachem, Habte Belete, Tilahun Belete Mossie, Azi Berzengi, Necip Capraz, Deniz Ceylan, Daniel Dukes, Aziz Essadek, Naved Iqbal, Laura Jobson, Einat Levy-Gigi, Antonia Lüönd, Chantal Martin-Soelch, Tanja Michael, Misari Oe, Miranda Olff, Helena Örnkloo, Krithika Prakash, Muniarajan Ramakrishnan, Vijaya Raghavan, Vedat Şar, Soraya Seedat, Georgina Spies, Vandhana SusilKumar, Dany Laure Wadji, Rachel Wamser-Nanney, Shilat Haim-Nachum, Ulrich Schnyder, Marie R. Sopp, Monique C. Pfaltz, Sarah L. Halligan

**Affiliations:** a Department of Psychiatry and Psychology, Bipolar and Depressive Disorders Unit, Hospital Clínic, Institute of Neurosciences (UBNeuro), Department of Medicine, Faculty of Medicine and Health Sciences, University of Barcelona (UB), Barcelona, Catalonia, Spain; b Fundació Clínic per a la Recerca Biomèdica (FCRB), Institut d'Investigacions Biomèdiques August Pi i Sunyer (IDIBAPS), Network Centre for Biomedical Research in Mental Health (CIBERSAM), Health Institute Carlos III (ISCIII), Barcelona, Catalonia, Spain; c Department of Psychology, University of Bath, Bath, UK; d Department of Psychology, University of the Bundeswehr Munich, Munich, Germany; e Department of Psychology and Social Work, Mid Sweden University, Östersund, Sweden; f Facultad de Medicina, Universidad de Piura, Lima, Peru; g Facultad de Medicina “San Fernando”, Universidad Nacional Mayor de San Marcos, Lima, Peru; h Department of Psychology, University of Zurich, Zurich, Switzerland; i Department of Psychiatry, College of Medicine and Health Sciences, Bahir Dar University, Bahir Dar, Ethiopia; j Department of Clinical Psychology and Psychological Therapies, University of East Anglia, Norwich, UK; k Private Practice, Istanbul, Turkey; l Department of Psychiatry, Koç University School of Medicine, Istanbul, Turkey; m Swiss Center for Affective Sciences, University of Geneva, Geneva, Switzerland; n Interpsy EA4432, University of Lorraine, Nancy, France; o Hôpitaux de Saint-Maurice, Saint-Maurice, France; p Department of Psychology, Jamia Millia Islamia, New Delhi, India; q School of Psychological Sciences, Turner Institute for Brain and Mental Health, Monash University, Clayton, Australia; r Faculty of Education, Bar-Ilan University, Ramat-Gan, Israel; s The Gonda Multidisciplinary Brain Research Center, Bar-Ilan University, Ramat-Gan, Israel; t Department of Adult Psychiatry and Psychotherapy, Psychiatric University Hospital Zurich, University of Zurich, Zurich, Switzerland; u Department of Psychology, University of Fribourg, Fribourg, Switzerland; v Division of Clinical Psychology and Psychotherapy, Saarland University, Saarbrücken, Germany; w Department of Neuropsychiatry, School of Medicine, Kurume University, Kurume, Japan; x Department of Psychiatry, Amsterdam Neuroscience & Public Health, Amsterdam UMC, University of Amsterdam, Amsterdam, The Netherlands; y ARQ National Psychotrauma Centre, Diemen, The Netherlands; z Department of Psychology, Eastern Michigan University, Ypsilanti, USA; aa Schizophrenia Research Foundation, Chennai, Tamil Nadu, India; ab Department of Psychiatry, Faculty of Medicine and Health Sciences, Stellenbosch University, Stellenbosch, South Africa; ac South African PTSD Programme of Excellence, Department of Psychiatry, Faculty of Medicine and Health Sciences, Stellenbosch University, Cape Town, South Africa; ad South African Medical Research Council / Stellenbosch University Genomics of Brain Disorders Research Unit, Department of Psychiatry, Stellenbosch University, Cape Town, South Africa; ae Kanchi Kamakoti CHILDS Trust Hospital (KKCTH), Chennai, Tamil Nadu, India; af Saveetha Medical College and Hospital, Saveetha Institute of Medical & Technical Sciences (SIMATS), Thandalam, Chennai, Tamil Nadu, India; ag Department of Educational and Counselling Psychology, McGill University, Montreal, Canada; ah Psychological Sciences Faculty, University of Missouri – St. Louis, St. Louis, Missouri, USA; ai Department of Psychiatry, Columbia University, New York, USA; aj School of Social Work, Tel Aviv University, Tel Aviv, Israel; ak Department of Consultation-Liaison Psychiatry and Psychosomatic Medicine, University Hospital, University of Zurich, Zurich, Switzerland; al Department of Psychiatry and Mental Health, University of Cape Town, Cape Town, South Africa

**Keywords:** PTSD, complex PTSD, social support, sex, gender, adults, TEPT, TEPT complejo, apoyo social, sexo, género, adultos

## Abstract

**Background:** Perceived social support is an established predictor of post-traumatic stress disorder (PTSD) after exposure to a traumatic event. Gender is an important factor that could differentiate responses to social support, yet this has been little explored. Symptoms of complex PTSD are also common following trauma but have been under-researched in this context. Large scale studies with culturally diverse samples are particularly lacking.

**Objectives:** In a multi-country sample, we examined: (a) gender differences in perceived social support and both posttraumatic stress symptom severity (PTSS) and complex posttraumatic stress symptom severity (CPTSS); (b) associations between social support and PTSS/CPTSS; and (c) the potential moderating role of gender in the relationship between perceived social support and trauma-related distress.

**Method:** A total of 2483 adults (*M_age_ *= 30yrs, 69.9% females) from 39 countries, who had been exposed to mixed trauma types, completed the Multidimensional Scale of Perceived Social Support and the International Trauma Questionnaire (which captures PTSS/CPTSS). Regression analyses examined associations between gender, perceived social support, and PTSS/CPTSS; and tested for gender by social support interactions in predicting PTSS/CPTSS scores. Models were adjusted for age and socioeconomic status.

**Results:** In our cross-country sample, females had greater PTSS/CPTSS than males (*B* = .23 [95% CI 0.16, 0.30], *p* < .001; *B* = .20 [0.12, 0.27], *p* < .001; respectively), but there was no evidence of gender differences in perceived social support (*B* = .05 [−0.05, 0.16], *p* = .33). For both genders, low perceived social support was associated with higher PTSS/CPTSS (females: *B* = −.16 [−0.20, −0.12], *p* < .001; *B* = −.27 [−0.30, −0.24], *p* < .001; respectively; males: *B* = −.22 [−0.29, −0.15], *p* < .001; *B* = −.31 [−0.36, −0.26], *p* < .001; respectively), and for PTSS only we found weak evidence that this association was stronger for males vs. females (*B* = .07 [0.04, 0.14, *p* = .04).

**Conclusion:** Individuals who feel more socially supported have lower trauma-related distress, and this association is similar in males and females. PTSD/CPTSD interventions may benefit from augmenting perceived social support, regardless of gender.

## HIGHLIGHTS

- In our multi-country sample, females show higher levels of (complex) posttraumatic stress symptom severity than males.
- There is no evidence of gender differences in perceived social support.
- Greater perceived social support is associated with lower (complex) posttraumatic stress symptom severity across both genders.

## Abstract (es)

**Antecedentes:** El apoyo social percibido es un predictor establecido del trastorno de estrés postraumático (TEPT) después de la exposición a un evento traumático. El género es un factor importante que podría diferenciar las respuestas al apoyo social, pero esto ha sido poco explorado. Los síntomas de (TEPTc) complejo también son comunes después de un trauma, pero no se han investigado lo suficiente en este contexto. En particular, faltan estudios a gran escala con muestras culturalmente diversas.

**Objetivos:** En una muestra de varios países, examinamos: (a) diferencias de género en el apoyo social percibido y tanto la gravedad de los síntomas de estrés postraumático (SEPT) como de los síntomas de estrés postraumático complejo (SEPTC); (b) las asociaciones entre apoyo social y SEPT/SEPTC, y, (c) el posible papel moderador del género en la relación entre el apoyo social percibido y el estrés traumático.

**Método:** Un total de 2.483 adultos (Media de edad = 30 años, 69.9% mujeres) de 39 países, que habían estado expuestos a distintos tipos de trauma completaron la Escala Multidimensional de Apoyo Social Percibido y el Cuestionario Internacional de Trauma (que captura SEPT/SEPTC). Mediante análisis de regresión se examinaron las asociaciones entre el género, el apoyo social percibido y SEPT/SEPTC y la interacción del género con el apoyo social para predecir las puntuaciones de SEPT/SEPTC. Los modelos se ajustaron por edad y estrato socioeconómico.

**Resultados:** En nuestra muestra transnacional, las mujeres tuvieron mayores SEPT/SEPTC que los hombres (*B* = .23 [95% CI 0.16, 0.30], *p* < .001; *B* = .20 [0.12, 0.27], *p* < .001; respectivamente), pero no se evidenciaron diferencias de género en el apoyo social percibido (*B* = .05 [−0.05, 0.16], *p* = .33). Para ambos géneros, el bajo apoyo social percibido se asoció con mayores SEPT/SEPTC (mujeres: *B* = −.16 [−0.20, −0.12], *p* < .001; *B* = −.27 [−0.30, −0.24], *p* < .001; respectivamente; hombres: *B* = −.22 [−0.29, −0.15], *p* < .001; B = −.31 [−0.36, −0.26], *p* < .001; respectivamente), y solo para los SEPT se encontró una evidencia débil que esta asociación se observó más fuerte para hombres que para mujeres (*B* = .07 [0.04, 0.14, *p* = .04).

**Conclusión:** Las personas que se sienten más apoyadas socialmente tienen un menor malestar relacionado con el trauma y esta asociación es similar en hombres y mujeres. Las intervenciones para TEPT/TEPTc pueden beneficiarse aumentando el apoyo social percibido, independientemente del género.

## 1. Introduction

In adults, sex and gender differences (Heidari et al., 2016)^1^ are clearly present in post-traumatic stress disorder (PTSD) incidence, with females being twice as likely to be diagnosed with PTSD than males (Olff et al., 2007; Tolin & Foa, 2008), although males seem to be at increased risk of trauma exposure (Carmassi et al., 2014). Females also report a higher PTSD symptom count than males (Carragher et al., 2016), particularly in the immediate aftermath of trauma, with some evidence that they may show a stronger recovery over time (Diamond et al., 2022; Hiscox et al., 2023). Gender effects are apparent even in samples where all individuals are exposed to the same type of traumatic event (Wade et al., 2016), meaning that they cannot be fully explained by differential vulnerability to experiencing certain types of trauma. Whilst biological, psychological, and social explanations for gender differences in posttraumatic stress symptom severity (PTSS) exist, there are no definitive conclusions as to why sex/gender differences are so prevalent in PTSD onset and PTSS (Zalta et al., 2021).

A key risk factor for PTSS is a lack of social support, with low perceived social support (i.e. how well supported or connected the individual feels) being associated with more severe PTSS (Blais et al., 2021; Christens et al., 2011). Perceived social support may provide protection following trauma by buffering stress, providing opportunities for continued engagement in rewarding activities, and potentially supporting beneficial disclosures of trauma-related memories and appraisals, simultaneously limiting avoidant coping (Shallcross et al., 2016). Consistent with this, a meta-analysis of longitudinal evidence has highlighted perceived social support as a negative predictor of later PTSD (Wang et al., 2021). Notably, reciprocal effects were also identified in the same analysis, whereby the presence of PTSD was equally predictive of lower subsequent social support (Wang et al., 2021), suggesting a negatively reinforcing cycle. Importantly, there is also evidence that perceived social support is related to treatment outcomes for PTSD, including enhancing patient engagement and adherence (Keller et al., 2010) and predicting stronger treatment responses (Price et al., 2018). Given these findings, exploring factors that may play a role in the relationship between perceived social support and PTSS can have critical implications for treating and managing PTSD.

Sex differences have been discovered in perceived social support levels, with women tending to report higher levels of support than men (Coventry et al., 2004). Gender role socialisation may prompt girls to foster and cherish social connections from a young age (Eccles et al., 2000) and potentially to place more value on social support, with evidence indicating that women take more reward and gratification from non-familial relationships than men (Osborne et al., 2008). After experiencing trauma, females are more likely than males to seek comfort in their social networks (Olff, 2017). Theoretically, therefore, social networks and perceived social support levels may be a greater predictor of mental well-being post-trauma in females *vs.* males. However, there has been limited research that directly addresses this question within a single sample, and two recent meta-analyses have yielded mixed findings. In a meta-analysis of studies examining associations between social support and PTSD, Zalta and collegues found moderately strong PTSD-social support associations and no evidence that this effect was influenced by sample sex distribution (Zalta et al., 2021). In contrast, a meta-analysis of longitudinal studies found that reciprocal longitudinal associations between social support and PTSS were moderated by gender (Wang et al., 2021). However, unexpectedly, predictive associations from social support to PTSS strengthened as the proportion of males in the sample increased, suggesting that men, not women, may benefit more. Importantly, whether social support and PTSD differ by gender at an individual study level is yet to be explored using a well-powered analysis.

A further gap in the evidence relates to our understanding of complex PTSD (CPTSD), which was recently included in the ICD-11 to capture disturbances of self-organisation (DSO: problems in affect regulation, negative self-concept, and relationship disturbances) that can occur alongside core PTSD symptoms in trauma survivors. To date, there is limited research on gender differences and associations with social support in CPTSD. In contrast to robust evidence of elevated rates of PTSD among women compared to men (Tolin & Foa, 2008), initial investigations on sex/gender differences in CPTSD have yielded mixed results (Lonnen & Paskell, 2024). Thus, a multi-country study found higher rates of CPTSD in females *vs.* males in a representative sample of US Americans, but no sex differences in representative samples from Ireland and Israel, or in a UK community sample, whereas sex differences in PTSD were present for all country groups (McGinty et al., 2021). In addition, in a representative Irish sample, the same researchers reported no sex effects for either PTSS or CPTSS in multivariate models including a range of predictors (McGinty et al., 2023). By contrast, in a sample of adolescents from Lithuania and Japan, female gender was associated with roughly two-times higher likelihood of a probable diagnosis of both PTSD and CPTSD (Kazlauskas et al., 2022). Notably, the interpretation of analyses of disorder prevalence is complicated by the fact that a diagnosis of CPTSD requires that the core PTSD criteria are also met, meaning that it is difficult to determine whether any sex/gender differences in CPTSD diagnoses are actually specific to complex disorder symptoms. Analyses separately examining the symptoms of PTSD and CPTSD could address this limitation.

Evidence in relation to associations between social support and complex posttraumatic stress symptom severity (CPTSS) is also mixed, despite the fact that difficulties maintaining relationships and feeling close to others are part of the ICD-11 CPTSD diagnostic criteria. Thus, in a sample of adults exposed to significant childhood adversity, Maercker et al. (2022) reported that low social support was associated with higher levels of both core PTSD symptoms and DSO symptoms of CPTSD, but associations for the latter were stronger and more robust (Maercker et al., 2022). By contrast, a study of a representative Irish sample found that low social support was associated with the core symptoms of PTSD but not with CPTSS, and that loneliness was related to both symptom sets but showed stronger associations with CPTSS (McGinty et al., 2023).

Studies of help-seeking samples have also yielded mixed findings with respect to CPTSD. A study of a Welsh clinical sample found limited evidence that either PTSS or CPTSS were associated with lower perceived social support (Simon et al., 2019), with the only robust negative association emerging for the relationship disturbances cluster of CPTSS. By contrast, an investigation of a Lithuanian clinical sample found that social support showed small associations with PTSD core symptoms only, not with CPTSS (Kvedaraite et al., 2021). Finally, in a sample of help-seeking refugees, a lack of social support was found to be associated with CPTSS only (Hecker et al., 2018). It remains to be investigated whether the influence of social support on CPTSS depends on gender.

Importantly, the evidence base as a whole is heavily biased towards Western countries, yet differing social contexts, family structures, norms, and values across countries can all moderate perceived social support levels (Bryant-Davis et al., 2011). In non-Western countries, sharing living quarters with extended family and shared financial obligations are more common than in Western countries (Wüsten & Lincoln, 2017) and such proximity can affect perceived social support levels (Scelza, 2011). Country-level factors such as whether countries have more collectivist cultures vs. individualistic cultures (Kim et al., 2006), or perceptions of the extent to which a country nurtures a safe and strong social context for its citizens (i.e. generalised trust) (Bi et al., 2021), can affect perceived social support through socialisation processes (Schneier, 2012). However, two recent meta-analyses have highlighted the limited representation of non-western countries (Wang et al., 2021; Zalta et al., 2021). Given that perceived social support may be culturally bound, it is critical to test the potential association of perceived social support on PTSS in samples drawn from diverse countries.

In a large multi-country sample of adults, we sought to confirm previous observations of gender differences in both perceived social support and PTSS, with females expected to score higher in both domains. We extended our analyses to also include CPTSS, as these are relatively common in trauma survivors but have been much less studied. We took a dimensional approach, allowing the full continuum of symptoms to be assessed separately for each of these two clusters. In addition, we tested associations between social support and both PTSS and CPTSS; and we further examined gender as a potential moderator of these associations. Here, based on previous observations that females may benefit more from social relationships than males (Osborne et al., 2008), we predicted that higher levels of perceived social support would be more strongly associated with lower PTSS/CPTSS in females relative to males.

## 2. Method

### 2.1. Design

We conducted a cross-sectional analysis of data from a pre-existing international dataset. The study was part of the Global Collaboration on Traumatic Stress initiative (https://www.global-psychotrauma.net/child-maltreatment) and study data have been made freely available via the Open Science Framework (https://osf.io/nxrfu/?view_only=25248e7fe4174f8b88cfe14c57e19e9d). Haim-Nachum et al. (2024) provides a full study protocol, detailing sampling characteristics and all assessments. Briefly, participants were recruited from multiple countries and completed indices of trauma, social functioning, and mental health.

### 2.2. Participants

Participants aged 18 and over were recruited from multiple countries, with a total of 3656 individuals partaking in the primary study (Haim-Nachum et al., 2024). Countries were selected with the aim of increasing cultural diversity and maximising socioeconomic variation. Instructions and questionnaires were offered in 12 languages: Afrikaans, Amharic, Arabic, English, French, German, Hebrew, Japanese, Spanish, Swedish, Turkish, and Xhosa. Participants were recruited from both student populations and members of the public using available online platforms for researchers from each country. Participants were included in the current analysis if they had experienced a traumatic event in their lifetime, based on their reporting of lifetime exposure to trauma on the Life Events Checklist (LEC) for DSM-5 (Weathers et al., 2013). Following data cleaning for suspicious responses (e.g. flatliners) and highly incomplete data (<10% of responses), there were 3466 participants in the final sample. Of these, 2507 reported lifetime exposure to trauma. We excluded 24 participants who did not identify as either male or female as numbers were too small for separate analyses, resulting in a sample of 2483. Finally, eight participants with missing covariate data were excluded from fully adjusted analyses, resulting in a sample of 2475.

### 2.3. Ethics

The original study obtained umbrella ethical approval from Saarland University (identification number 21-07), with local ethical approval also acquired in countries that required a separate ethics application. Prior to starting the study, participants provided full informed consent.

### 2.4. Measures

***Gender:*** Participants were asked ‘What is your sex?’ with answer options of ‘Female’, ‘Male’, or ‘I do not identify with either.’ Due to the small proportion of individuals reporting ‘I do not identify with either’ (*n* = 24), these participants were not included in the analysis, and a binary Female/Male variable was used in all models. As this question pertains to participants’ self-identification rather than sex assigned at birth, it most accurately captures gender rather than biological sex.

***Demographic characteristics:*** Participants provided their birth year which was used to determine age. SES was indexed through participants placing themselves on a 10-rung ladder representing ‘where people stand in the country you currently live in’, with the top rung representing best off and the bottom the worst off, in terms of money, education and job status.

***(Complex) Posttraumatic symptom severity:*** The International Trauma Questionnaire (ITQ) (Cloitre et al., 2018) was used to assess ICD-11 PTSS and CPTSS (Gelezelyte et al., 2022). This 18-item self-report scale has two major subscales with three symptom clusters in each: (1) PTSD symptoms, including re-experiencing, Avoidance, and Sense of current threat, and (2) symptoms of CPTSD, including Affective dysregulation, Negative self-concept, and Disturbances in relationships (Maercker et al., 2013). The ITQ asks participants to answer questions in relation to a specific traumatic event using a 5-point Likert scale and symptoms are each rated from 0 = ‘not at all’ to 4 = ‘extremely’, with six items each measuring PTSD/CPTSD symptoms. The ITQ has established reliability and validity (Cloitre et al., 2021; Frost et al., 2022; Gelezelyte et al., 2022) (in the current study PTSS subscale Cronbach’s α = .84, CPTSS α = .87).

***Perceived social support****:* The Multidimensional Scale of Perceived Social Support (MSPSS) (Zimet et al., 1988) was used to assess perceived support from a significant other, family, and friends. The MSPSS consists of 12 items each rated from 1 = ‘very strongly disagree’ to 7 = ‘very strongly agree’. The MSPSS shows high levels of internal reliability and factorial validity across various demographics (Dahlem et al., 1991; Zimet et al., 1990) and diverse cultures (Wongpakaran et al., 2011; Zhou et al., 2015; Zimet et al., 1990). Cronbach’s alpha for the total scale in the current study was α = .93.

***Statistical analysis*** The association of perceived social support with PTSD symptoms was assessed using negative binomial regression modelling. The negative binomial regression model is a type of generalised linear model used for count data, particularly when a variable displays overdispersion. It can be considered an extension of the Poisson regression model, which assumes that the mean and the variance of the count data are equal. PTSD outcome variables showed significant left skew and overdispersion, and were not amenable to transformation. A negative binomial model was therefore used for all analyses. Two dimensions of PTSD symptoms were assessed: PTSS and CPTSS. Models were adjusted for the following covariates: age, gender (females vs. males), and SES based on a review of previous work (Zalta et al., 2021). The potentially moderating effects of gender (female vs. male) were also examined. First, the association of gender with PTSD outcomes was explored, with all models adjusted for age and SES. Second, the models described in the main analyses were rerun stratifying on gender to explore if effect estimates and 95% confidence intervals (CIs) differed between groups. Finally, interaction analysis was conducted to assess whether there were differences in the association between perceived social support and PTSS across categories of gender. Specifically, analyses were conducted to understand if gender was a moderator that changed the direction and/or strength of the relationship between the exposure and outcome variables. This was done by introducing an interaction term between perceived social support and gender in the models described above. The interaction coefficient, CIs and *p*-value were appraised to assess whether the relationship between perceived social support and PTSD symptoms varies between males and females. The study sample was skewed in terms of participant age. In a sensitivity analysis we therefore reran all analyses with age included as a categorical covariate (18-24 years (*N *= 1202, 48.57%), 25–34 years (*N* = 582, 23.52%), 35–44 years (*N* = 289, 11.68%), 45–54 years (*N* = 196, 7.92%), > 55 years (*N* = 206, 8.32%)) to enable comparison to the main analyses and assess the impact of age distribution on our findings.

## 3. Results

### 3.1. Participant characteristics

The final sample comprised 2483 participants from 39 different countries, with the highest proportions from South Africa (14.98%), India (14.70%), Switzerland (13.65%), Germany (9.34%), and Sweden (6.28%) (Supplementary Table 2 provides full distribution of participants by current country of residence). Mean years of education in the study sample was 15.5 years, indicating a relatively well-educated sample in which the average age of leaving education exceeds 18-years (e.g. university level). Responses to the LEC demonstrated that the most frequently experienced traumatic events were learning about the sudden violent death (900 participants; 36.25%), accidental death (878; 35.36%), or life-threatening illness or injury (888; 35.76%) of a loved one. In addition, 998 participants (40.19%) reported experiencing another ‘very stressful event or experience’ not listed in the checklist (see Supplementary Table 1 for full details of the traumatic events reported). Within this trauma-exposed sample, 10.96% of males and 19.31% of females met the criteria for PTSD based on established cut-offs for the ITQ, and 5.35% of males and 11.12% of females met the criteria for CPTSD. See Table 1 for sample sociodemographic and clinical characteristics.

**Table 1.** Sociodemographic and clinical characteristics of the trauma-exposed sample (*N* = 2483).

	Females *n* = 1735, 69.9%	Males *n* = 748, 30.1%	Total *N* = 2483
**Sociodemographic variables**			
Age, Mean in years *(SD)*	29.0 (12.6)	34.0 (14.2)	30.5 (13.3)
Education, Mean in years *(SD)*	15.4 (4.0)	15.8 (4.0)	15.5 (4.0)
SES, Median *(range)*	6 (1–10)	6 (1–10)	6 (1–10)
**Clinical variables**	** **	** **	
ITQ, PTSD symptoms, Mean *(SD)*			
Re-experiencing	2.01 (2.14)	1.54 (1.84)	1.87 (2.06)
Avoidance	2.76 (2.56)	1.88 (2.26)	2.50 (2.51)
Sense of Threat	2.65 (2.61)	1.94 (2.29)	2.44 (2.54)
Total	7.42 (6.19)	5.36 (5.43)	6.80 (6.05)
ITQ, CPTSD symptoms, Mean *(SD)*			** **
Affective dysregulation	2.99 (2.03)	2.29 (1.95)	2.78 (2.03)
Negative self-concept	2.24 (2.45)	1.64 (2.27)	2.06 (2.45)
Disturbed relationships	2.74 (2.50)	2.36 (2.39)	2.63 (2.43)
Total	7.98 (6.03)	6.29 (5.78)	7.47 (6.01)
MSPSS, Mean *(SD)*			
Significant Other	4.55 (1.29)	3.97 (1.30)	4.38 (1.32)
Family	5.15 (1.59)	5.23 (1.50)	5.17 (1.60)
Friends	5.33 (1.47)	5.13 (1.46)	5.27 (1.47)
Total	5.37 (1.24)	5.26 (1.27)	5.33 (1.25)

Note*.* SES = Socioeconomic Status as social class defined with the Social ladder rank; ITQ = International Trauma Questionnaire; PTSD = posttraumatic stress disorder; CPTSD = complex posttraumatic stress disorder; MSPSS = Multidimensional Scale of Perceived Social Support.

### 3.2. Gender differences in posttraumatic distress and perceived social support

We used regression analyses to test for gender differences in PTSS and CPTSS (both measured by the ITQ); and in perceived social support (MSPSS). All models were adjusted for age and SES. As expected, females vs. males showed higher PTSS (*B* = .23, 95% CI = 0.16, 0.30, *p* < .001) and CPTSS (*B* = .20, 95% CI = 0.12, 0.27, *p* < .001). However, we found no evidence of gender differences in perceived social support in the adjusted regression model (*B* = .05, 95% CI = −0.05, 0.16, *p* = .33). See Table 2 for mean scores for all scales presented by gender.

**Table 2.** Prediction of symptoms of PTSD/complex PTSD by perceived social support, gender, and their interaction; models adjusted for age; Analyses conducted in the full trauma-exposed sample.

		Posttraumatic stress symptoms			Complex PTSD symptoms		
		*B* (SE)	95% CI	*p*	*B* (SE)	95% CI	*p*
**Full sample**	Gender	−.08 (.19)	−.46, .30	.68	.02 (.15)	−.05, −.02	.91
*N* = 2475	Perceived social support	−.23 (.03)	−.29, −.17	<.001	−.31 (.02)	−.36, −.26	<.001
	Gender × perceived social support	.07 (.04)	.004, .14	.04	.04 (.03)	−.02, .10	.16
	SES	−.05 (.01)	−.08, −.02	<.001	−.04 (.01)	−.05, −.02	<.001
	Age	−.01 (.002)	−.02, −.01	<.001	−.02 (.001)	−.02, −.01	<.001
**Females only**	Perceived social support	−.16(.02)	−.20, −.12	<.001	−.27 (.02)	−.30, −.24	<.001
*n* = 1728	SES	−.03 (.01)	−.06, −.003	.03	−.03 (.001)	−.06, −.01	.002
	Age	−.02 (.002)	−.02, −.01	<.001	−.02 (.001)	−.02, −.01	<.001
**Males only**	Perceived social support	−.22 (.03)	−.29, −.15	<.001	−.31 (.03)	−.36, −.26	<.001
*n* = 747	SES	−.07 (.02)	−.12, −.03	.002	−.04 (.02)	−.08, −.004	.03
** **	Age	−.01 (.003)	−.02, −.01	<.001	−.02 (.002)	−.02, −.01	<.001

Note*.* SES = Socioeconomic Status as social class defined with the Social ladder rank; PTSD = posttraumatic stress disorder; CI = Confidence Interval; SE = Standard Error.

### 3.3. Potential moderation of associations between PTSS/CPTSS and perceived social support by gender

We used regression modelling to examine the prediction of PTSS/CPTSS by gender, MSPSS scores, and their interaction, adjusting for age and SES. We found a robust, inverse association between perceived social support and PTSS. In addition, we found weak evidence that the relationship between MSPSS and PTSS scores was moderated by gender (*B *= .07, 95% CI = 0.004, 0.14, *p* = .04). Gender-stratified analyses showed a slightly stronger inverse association between MSPSS and PTSS scores in males (*B *= −.22, 95% CI = −0.29, −0.15, *p* < .001) vs. females (*B* = −.16 95% CI = −0.20, −0.12, *p* < .001). When this analysis was repeated with CPTSS as the outcome the findings were similar, with a strong inverse association between MSPSS and CPTSS scores. However, there was no evidence of moderation by gender. Full model results are presented in Table 2.

### 3.4. Sensitivity analysis

To evaluate the influence of the skewed age covariate on our results, we reran all models with the inclusion of a categorical age variable. We observed consistent direction and strength of associations among variables across models, with minimal variations in effect estimates and CIs throughout. Please refer to Supplementary Table 3 for the full reported findings.

## 4. Discussion

Here, in a large, cross-country convenience sample, we explored gender differences in perceived social support and PTSS/CPTSS, and gender as a moderating variable in the relationship between perceived social support and PTSS/CPTSS. We found that whilst females showed higher levels of PTSS/CPTSS than males, there were no gender differences in perceived social support. Moreover, greater perceived social support was robustly associated with lower PTSS/CPTSS across genders, but for PTSS this association was marginally stronger for males vs. females.

Consistent with a large, extant literature, trauma-exposed females in our sample demonstrated greater PTSS than males (Ainamani et al., 2020; Tolin & Foa, 2008). Importantly, in our multiple country sample, we extended these observations by identifying similar gender differences in the symptoms of CPTSD. The latter have been little studied in relation to gender thus far, and a consistent pattern of findings is yet to emerge, with the available evidence variously failing to identify sex differences in CPTSS in multivariate models (McGinty et al., 2023), providing only partial evidence of sex effects in relation to CPTSD prevalence (McGinty et al., 2021), or showing gender differences in CPTSD that are similar to those reported for PTSD (Kazlauskas et al., 2022). From a conceptual standpoint, it is conceivable that due to the cumulative, often prolonged, and early-life onset of traumas associated with CPTSD, sex-specific vulnerabilities may be less significant. Nonetheless, our findings suggest that females may be more vulnerable to CPTSS, implying that similar vulnerabilities to those observed in PTSD can be presumed. To make definitive statements, further large-scale studies are needed to examine the relationship between both gender and sex and CPTSD/CPTSS and to probe potential underlying mechanisms (e.g. presence and severity of comorbid symptoms, self-blame/criticism, avoidant coping strategies) (Whiffen & Macintosh, 2005) and protective factors that may buffer stress (Shallcross et al., 2016) or promote resilience (Fares-Otero et al., 2023).

Whilst there is some evidence that females have greater perceived social support than males (Coventry et al., 2004), and that there are gender differences in the structure of social support in non-traumatised samples (Matud et al., 2003), little research has explored whether this gender difference endures post-trauma exposure (Zalta et al., 2021). We found no evidence of gender differences in perceived social support in our large, trauma-exposed sample, and no evidence to support the notion that males receive less gratification from social networks than females (Osborne et al., 2008), at least as it applies to posttrauma mental health. Indeed, our observation of a slightly stronger inverse association between social support and PTSS in males *vs.* females is consistent with the conclusions of a prior meta-analysis, which found that longitudinal associations between social support and PTSS were stronger in samples with a higher proportion of males (Wang et al., 2021).

The general lack of gender differences in levels of social support in our sample could be due to trauma exposure either differentially eroding perceived social support among females or enhancing support among males. It could also be a function of our sample, which was skewed towards young adults. With social activities increasingly occurring online, particularly for younger age groups, societal changes in how social support is accessed could have impacted the extent of gender differences. A study examining the relationship between use of Facebook, a popular online social network site, and the formation and maintenance of social capital demonstrated that young adult males have at least as many Facebook friends as females (Ellison et al., 2007). On the other hand, a more recent meta-analysis assessing the presence and magnitude of gender differences in social support on social network sites found that females provide, and to some extent, also receive more social support either offline or online than do males (Tifferet, 2020). The way in which social support is accessed across genders in relation to adverse experiences warrants further exploration given this changing landscape.

Our findings reaffirm that males and females may each benefit from increased social support in their psychological responses to trauma exposure, including in relation to both PTSD and CPTSD. Existing psychological and pharmacological interventions (Coventry et al., 2020; Purnell et al., 2021) should attend to the possible benefits of simultaneously augmenting social support in both males and females with PTSD/CPTSD as part of the recovery process. For example, interventions could involve family members and/or partners, including psychoeducation regarding PTSD/CPTSD and support provision; or behavioural components could be included to introduce activities targeting increased social interactions. Of course, our cross-sectional data preclude assumptions about causal direction, and it is also likely that both males and females experience impairments in their perceived levels of social support as a consequence of symptoms of PTSD/CPTSD. Future research should examine perceived social support as a potential mediator of recovery or an intervention outcome in order to disentangle these possibilities. Nonetheless, given the general importance of good social support for wellbeing, the inverse association between perceived social support and PTSD/CPTSD across genders warrants clinical attention regardless of causal direction.

As a further step, it will be necessary for future research to examine in more depth whether there are differences in social deficits and needs between PTSD and CPTSD. The limited existing literature on CPTSD suggests that interpersonal factors may be important (Hecker et al., 2018; Kvedaraite et al., 2021; Maercker et al., 2022; Simon et al., 2019), for example, with loneliness potentially playing a significant role (Kazlauskas et al., 2022; Kvedaraite et al., 2021; McGinty et al., 2023). Consideration of how social factors relate to the DSO criterion of *disturbances in relationships* will be particularly important. Direct comparisons should be made regarding the influences of social factors such as perceived social support and loneliness on PTSD/PTSS vs. CPTSD/CPTSS, considering bidirectional relationships and potential gender differences. This will allow psychotherapeutic treatment to be more precisely tailored to the specific disorder.

Major advantages of the current study include the large sample size that provided high statistical power and the use of a multi-country sample. Utilising a diverse representation of individuals showed that gender differences in PTSS and CPTSS are robust in a multi-country sample and that globally, higher perceived social support is associated with lower (complex) posttraumatic distress for both males and females. Our study, therefore, can inform the development of PTSD/CPTSD interventions internationally, with our findings applicable to both males and females in both Western and non-Western countries.

There are several limitations to consider when interpreting the results of this study. First, females and young adults, particularly university students, were over-represented in our sample. Students may have larger peer networks and less contact with established family networks (Boals et al., 2020) than middle-aged adults; this could skew MSPSS scores and the strength of the associations we discovered. However, a recent critical review (*N* = 17496) indicates that trauma exposure, severity and type, PTSD symptom clusters, PTSS, and associations with perceived social support and comorbidity rates of PTSD do not significantly vary between university and non-university samples (Boals et al., 2020). As such, it is likely that our findings are more widely generalisable. Second, although we included multiple countries in our sample we were not able to look at country level differences; this could be a useful avenue for future exploration. Third, we were only able to obtain cross-sectional data, and cannot establish causation between perceived social support and PTSS/CPTSS. There is evidence of a bidirectional relationship between social support: individuals with PTSD or CPTSD may experience a complex feedback loop between social support and symptom severity, with changes in one domain leading to changes in the other (Kaniasty & Norris, 2008; Zalta et al., 2014). Future research should examine the relationship between social support and PTSS/CPTSS longitudinally, testing whether social support may influence the stronger posttrauma recovery observed in females *vs.* males, including both spontaneous symptom recovery (Diamond et al., 2022) and treatment-related symptom decline (Hiscox et al., 2023). Fourth, our analyses did not take account of the possible presence of commonly co-occurring mental health conditions, such as major depressive disorder or anxiety disorders, which are also linked to lower perceived social support (Wang et al., 2018). Females with PTSD have been found to show higher rates of comorbid anxiety disorders compared to males in a US representative sample (Pietrzak et al., 2011). As such, comorbid mental disorders represent potentially confounding variables. It will be important to address this possibility in future work, with transdiagnostic data collected on mental health. Finally, it is also critical to note that our brief survey instrument did not adhere to best practise guidelines for capturing gender-identification *vs.* biological sex. The use of gold-standard instruments and the enrichment of samples for participants who do not identify with their sex assigned at birth, is needed to provide a fuller understanding of the roles of gender and sex in relation to PTSD/CPTSD.

## 5. Conclusion

To conclude, this study investigated gender differences in perceived social support and PTSS/CPTSS in a cross-country sample of adults. Additionally, gender was examined as a moderating variable in the relationship between perceived social support and posttraumatic distress. Females showed higher PTSS/CPTSS than males and an inverse association was found between perceived social support and posttraumatic distress that did not vary substantially as a function of gender. Future research should uncover the potentially complex relationship between perceived social support and PTSS/CPTSS in longitudinal samples, to establish the directionality of this relationship, underlying mechanisms, differences between PTSD and CPTSD, and the true role of gender.

## Supplementary Material

Supplemental Material

## Data Availability

The data that support the findings of this study are available from OSF at 10.31234/osf.io/nvejb.

## Note

1 Note that sex refers to biological attributes that are associated with physical and physiological features such as chromosomes, hormone levels, and reproductive/sexual anatomy, while gender refers to socially constructed roles, behaviours, expressions, and identities (Heidari et al., 2016). When discussing the evidence base, we used the terms reported by the authors, whilst noting that this distinction has often not been made clear.
