# Correction

**DOI:** 10.1080/20008066.2025.2498309

**Published:** 2025-06-06

Social support and (complex) posttraumatic stress symptom severity: does gender matter?

Natalia E. Fares-Otero, Tamsin H. Sharp, Stefanie R. Balle, Sarah M. Quaatz, Eduard Vieta,

Fredrik Åhs, Antje-Kathrin Allgaier, Adrián Arévalo, Rahel Bachem, Habte Belete,

Tilahun Belete Mossie, Azi Berzengi, Necip Capraz, Deniz Ceylan, Daniel Dukes, Aziz Essadek, Naved Iqbal, Laura Jobson, Einat Levy-Gigi, Antonia Lüönd, Chantal Martin-Soelch, Tanja Michael, Misari Oe, Miranda Olff, Helena Örnkloo, Krithika Prakash,

Muniarajan Ramakrishnan, Vijaya Raghavan, Vedat Şar, Soraya Seedat, Georgina Spies,

Vandhana SusilKumar, Dany Laure Wadji, Rachel Wamser-Nanney, Shilat Haim-Nachum,

Ulrich Schnyder, Marie R. Sopp, Monique C. Pfaltz and Sarah L. Halligan

*European Journal of Psychotraumatology*

https://doi.org/10.1080/20008066.2024.2398921

When the article was first published online, author Shreelakshmi Karthikeyan was omitted from the author list in error.

These errors have been corrected.

