# Supplementary material for: Social support and (complex) posttraumatic stress symptom severity: does gender matter?
Source: Eur J Psychotraumatol. 2024 Oct 15;15(1):2398921. doi: 10.1080/20008066.2024.2398921 (PMC11486103; doi:10.1080/20008066.2024.2398921)
Supplement: Supplemental Material [file ZEPT_A_2398921_SM4873.docx]

**Supplementary Table 1.** *Distribution of participant responses to exposure to traumatic events as categorised by the Life Events Checklist (LEC)*

|  |  |  |  |  |  |  |  |  |  |  |
| --- | --- | --- | --- | --- | --- | --- | --- | --- | --- | --- |
| **Event; n (%)** | **Happened to me** | | **Witnessed it** | | **Learned about it** | | **Part of my job** | | **Does not apply** | |
| 1. Natural disaster (for example, flood, hurricane, tornado, earthquake) | 575 | (23.16) | 440 | (17.72) | 710 | (28.59) | 33 | (1.33) | 1046 | (42.13) |
| 2. Fire or explosion | 239 | (9.63) | 553 | (22.27) | 704 | (28.35) | 60 | (2.42) | 1131 | (45.55) |
| 3. Transportation accident (for example, car accident, boat accident, train wreck, plane crash) | 693 | (27.91) | 700 | (28.19) | 896 | (36.09) | 66 | (2.66) | 609 | (24.53) |
| 4. Serious accident at work, home, or during recreational activity | 296 | (11.92) | 435 | (17.52) | 750 | (30.21) | 89 | (3.58) | 1178 | (47.44) |
| 5. Exposure to toxic substance (for example, dangerous chemicals, radiation) | 113 | (4.55) | 91 | (3.66) | 384 | (15.47) | 78 | (3.14) | 1915 | (77.12) |
| 6. Physical assault (for example, being attacked, hit, slapped, kicked, beaten up) | 593 | (23.88) | 541 | (21.79) | 756 | (30.45) | 81 | (3.26) | 1007 | (40.56) |
| 7. Assault with a weapon (for example, being shot, stabbed, threatened with a knife, gun, bomb) | 196 | (7.89) | 212 | (8.54) | 634 | (25.53) | 70 | (2.82) | 1552 | (62.51) |
| 8. Sexual assault (rape, attempted rape, made to perform any type of sexual act through force or threat of harm) | 414 | (16.67) | 92 | (3.71) | 788 | (31.74) | 76 | (3.06) | 1336 | (53.81) |
| 9. Other unwanted or uncomfortable sexual experience | 786 | (31.66) | 176 | (7.09) | 721 | (29.04) | 77 | (3.10) | 1128 | (45.43) |
| 10. Combat or exposure to a war-zone (in the military or as a civilian) | 107 | (4.31) | 110 | (4.43) | 488 | (19.65) | 50 | (2.01) | 1840 | (74.10) |
| 11. Captivity (for example, being kidnapped, abducted, held hostage, prisoner of war) | 46 | (1.85) | 42 | (1.69) | 361 | (14.54) | 32 | (1.29) | 2062 | (83.04) |
| 12. Life-threatening illness or injury | 315 | (12.69) | 764 | (30.77) | 888 | (35.76) | 114 | (4.59) | 880 | (35.44) |
| 13. Severe human suffering | 240 | (9.67) | 522 | (21.02) | 637 | (25.65) | 148 | (5.96) | 1269 | (51.11) |
| 14. Sudden violent death (for example, homicide, suicide) | 74 | (2.98) | 323 | (13.01) | 900 | (36.25) | 129 | (5.20) | 1258 | (50.66) |
| 15. Sudden accidental death | 107 | (4.31) | 423 | (17.04) | 878 | (35.36) | 99 | (3.99) | 1155 | (46.52) |
| 16. Serious injury, harm, or death you caused to someone else | 79 | (3.18) | 110 | (4.43) | 195 | (7.85) | 54 | (2.17) | 2119 | (85.34) |
| 17. Any other very stressful event or experience | 998 | (40.19) | 474 | (19.09) | 517 | (20.82) | 124 | (4.99) | 949 | (38.22) |

**Supplementary Table 2.** *Distribution of participants by current country of residence (N = 2483).*

| **Country** | **Frequency (%)** | |
| --- | --- | --- |
| South Africa | 372 | (14.98) |
| India | 365 | (14.70) |
| Switzerland | 339 | (13.65) |
| Israel | 238 | (9.59) |
| Germany | 232 | (9.34) |
| Sweden | 161 | (6.48) |
| Turkey | 147 | (5.92) |
| France | 124 | (4.99) |
| UK | 121 | (4.87) |
| Iraq | 85 | (3.42) |
| Cameroon | 78 | (3.14) |
| Japan | 66 | (2.66) |
| U.S. | 34 | (1.37) |
| Spain | 26 | (1.05) |
| Peru | 23 | (0.93) |
| Ethiopia | 20 | (0.81) |
| Australia | 14 | (0.56) |
| Namibia | 4 | (0.16) |
| Zimbabwe | 3 | (0.12) |
| Austria | 3 | (0.12) |
| Liechtenstein | 3 | (0.12) |
| Netherlands | 2 | (0.08) |
| Brazil | 2 | (0.08) |
| Lesotho | 2 | (0.08) |
| Kuwait | 2 | (0.08) |
| Chile | 2 | (0.08) |
| Not reported | 2 | (0.08) |
| Canada | 1 | (0.04) |
| Kenya | 1 | (0.04) |
| Mozambique | 1 | (0.04) |
| Greece | 1 | (0.04) |
| Thailand | 1 | (0.04) |
| Iran | 1 | (0.04) |
| Italy | 1 | (0.04) |
| Norway | 1 | (0.04) |
| Philippines | 1 | (0.04) |
| Colombia | 1 | (0.04) |
| Mexico | 1 | (0.04) |
| Uruguay | 1 | (0.04) |
| Kurdistan | 1 | (0.04) |
| Total | 2483 | (100.00) |

**Supplementary Table 3.** *Prediction of symptoms of PTSD/complex PTSD by perceived social support and their interaction; models adjusted for a categorical variable of age, SES (N =2475).*

|  |  | **Posttraumatic stress symptoms** | | |  | **Complex PTSD symptoms** | | |
| --- | --- | --- | --- | --- | --- | --- | --- | --- |
|  |  | *B* (SE) | 95% CI | *p* |  | *B* (SE) | 95% CI | *p* |
| **Full Sample** | Perceived social support | -.23 (.03) | -.29, -.17 | <.001 |  | -.32 (.02) | -.36, -.27 | <.001 |
|  | Gender x perceived social support | .07 (.04) | .004, .14 | .04 |  | .04 (.03) | -.02, .10 | .16 |
| **Females only** | Perceived social support | -.16 (.02) | -.20, -.12 | <.001 |  | -.27 (.02) | -.30, -.24 | <.001 |
| **Males only** | Perceived social support | -.22 (.03) | -.29, -.16 | <.001 |  | -.32 (.03) | -.37, -.26 | <.001 |

***Note****.* SES = Socioeconomic Status as social class defined with the Social ladder rank; PTSD = posttraumatic stress disorder; CI = Confidence Interval.
